# 215 The Effectiveness of Digital Tools in Physical Activity Interventions for Individuals with Severe Mental Illness: A Systematic Literature Review

**DOI:** 10.1093/eurpub/ckae114.187

**Published:** 2024-09-26

**Authors:** Shannon Aisling Forde, Tara Coppinger, Susan Rea, Sinéad Hanrahan

**Affiliations:** Department of Sports, Leisure & Childhood Studies, Munster Technological University, Bishopstown, Ireland; Department of Sports, Leisure & Childhood Studies, Munster Technological University, Bishopstown, Ireland; Nimbus Centre, Munster Technological University, Bishopstown, Ireland; Library, Munster Technological University, Bishopstown, Ireland

## Abstract

**Purpose:**

Individuals with a severe mental illness (SMI) have lower physical activity (PA) levels and higher sedentary behaviour (SB) than the general population. As a result, they have an elevated risk of cardiovascular and metabolic disease. Participating in PA benefits SMI by improving symptoms, cardiorespiratory fitness, social connectedness, and motivation; helping to reduce bodyweight and SB.

Despite the acknowledged benefits of PA, there are numerous barriers to engagement and participation rates remain low. The integration of a digital tool/s may mitigate these barriers and help increase the PA levels of individuals with SMI. This review aims to determine whether digital tool/s are effective at promoting PA in this population.

**Methods:**

A systematic literature review (SLR) was undertaken in March 2023. A search string was developed around key concepts of SMI, PA, and digital interventions. Seven databases were searched. Additional records obtained via alerts from databases were added between March-September 2023.

Returned records were exported to Rayyan. Duplicates were removed and the remaining articles were divided between the four-member team for title/abstract screening. Full-text screening followed, and quality checks were completed by the team via a standard checklist created on Google Forms. Articles selected for review were exported as a list into Microsoft Excel.

**Results:**

Database searches yielded 1,730 articles, with 24 eligible for inclusion.

Eleven studies found PA to increase from participation in a PA intervention that incorporated a digital tool. Walking was the most common form of PA (n = 13) and accelerometery (n = 13) was the most popular digital tool. Twice-weekly PA participation was most frequent. Moderate-vigorous PA was more effective than light PA at promoting PA. Supervised (n = 20) and group (n = 16) interventions were more successful in increasing PA. Community settings (n = 9) was the most common location for interventions. Few studies (n = 5) recorded follow-up data to ascertain if the intervention was effective long-term.

**Conclusion:**

Overall, this review found digital tools are effective at promoting PA among SMI individuals. Future PA interventions aimed at this population should consider incorporating this technology in their design. However, more follow-up studies are needed to ascertain whether these tools can sustain PA long-term.

**Funding:**

ADVANCE CRT

